# Construction and validation of a nomogram model for the differential diagnosis of primary testicular benign and malignant tumors

**DOI:** 10.1038/s41598-026-47759-1

**Published:** 2026-04-12

**Authors:** Chang-guo Wang, Song-chao Li, Hao He, Hao-xiang Jin, Zhan-kui Jia

**Affiliations:** 1 Department of Urology, The First Affiliated Hospital of Zhengzhou University, Zhengzhou, 450052 China; 2 Department of Urology, The First Affiliated Hospital of Henan Medical University, Xinxiang, China

**Keywords:** Testicular tumors, Differential diagnosis, Nomogram, Cancer, Diseases, Oncology, Urology

## Abstract

Testicular tumors often pose a significant threat to the health of young adult males, and inappropriate radical orchiectomy imposes a heavy physical and psychological burden on many patients.In order to accurately distinguish testicular benign and malignant tumors prior to treatment, we retrospectively enrolled 298 patients with testicular tumors treated at the First Affiliated Hospital of Zhengzhou University between June 2016 and June 2024, all of whom met the inclusion and exclusion criteria. Using a random number method, the patients were divided into a training set (209 cases, 70%) and an internal validation set (89 cases, 30%). Additionally, 55 patients from two different medical centers, treated between January 2019 and January 2024 and meeting the same criter-ia, were included as an external validation set. Tumor size (OR = 2.70, 95% CI: 1.80–4.05, *P* < 0.001), boundary (OR = 3.99, 95% CI: 1.34–11.86, *P* = 0.013), AFP (OR = 7.49, 95% CI: 1.92–29.25, *P* = 0.004), hCG (OR = 5.21, 95% CI: 1.30–20.95, *P* = 0.020), and CDFI (Grade II: OR = 4.23, 95% CI: 1.35–13.26, *P* = 0.014; Grade III: OR = 16.26, 95% CI: 3.68–71.83, *P* < 0.001) were identified as five independent discriminative factors for distinguishing benign from malignant testicular tumors. A nomogram model incorporating these factors was constructed and demonstrated favorable performance. The model was deployed online as a dynamic nomogram (https://nomogram98.shinyapps.io/dynnomapp/).

## Introduction

Testicular tumors are relatively rare but represent the most common solid malignancy among young men. Globally, the incidence of these tumors has been rising, particularly among Caucasian males aged 14 to 44 years, with higher rates reported in European countries such as Denmark and Norway^1^. The majority of primary testicular tumors are malignant, and germ cell tumors (GCTs) account for approximately 95% of these cases. Advances in platinum-based chemotherapy and surgical techniques have made testicular cancer one of the most curable malignancies, with a cure rate of about 95%. However, post-treatment complications—including impaired fertility, metabolic syndrome, and psyc-hological sequelae—continue to pose significant challenges and contribute to an increasing disease burden^2^.

Given the substantial differences in treatment and prognosis between benign and malignant testicular tumors, accurate preoperative discrimination is essential. Such differentiation helps avoid unnecessary radical orchiectomy (RO), faciitates organ-preserving strategies, and enables personalized management. However, distinguishing between benign and malignant testicular lesions remains challenging due to overlapping clinical, imaging, and laboratory findings. Currently, preoperative diagnosis largely depends on clinical experience, and cases still occur in which pathology reveals benign lesions after RO. There is a clear need for an integrated, easy-to-use predictive tool that combines multiple relevant parameters to aid clinical decision-making.

Nomograms have been widely adopted for discriminating benign from malignant conditions in other cancers. For example, Hu et al.^3^ developed a nomogram incorporating clinical and tumor scoring features to differentiate thyroid nodules, while Winarno et al.^4^ built a predictive model using serum biomarkers for ovarian tumor classification. These studies demonstrated that nomograms offer high accuracy and clinical utility. Nevertheless, a robust nomogram for discriminating testicular tumors is still lacking. This study aims to identify independent discriminative factors from routine clinical parameters, construct a corresponding nomogram model, and evaluate its discrimination, calibration, and clinical usefulness through internal and external validation. The ultimate goal is to provide a reliable and practical tool for preoperative differentiation of benign and malignant testicular tumors.

## Materials and methods

**Fig. 1:**
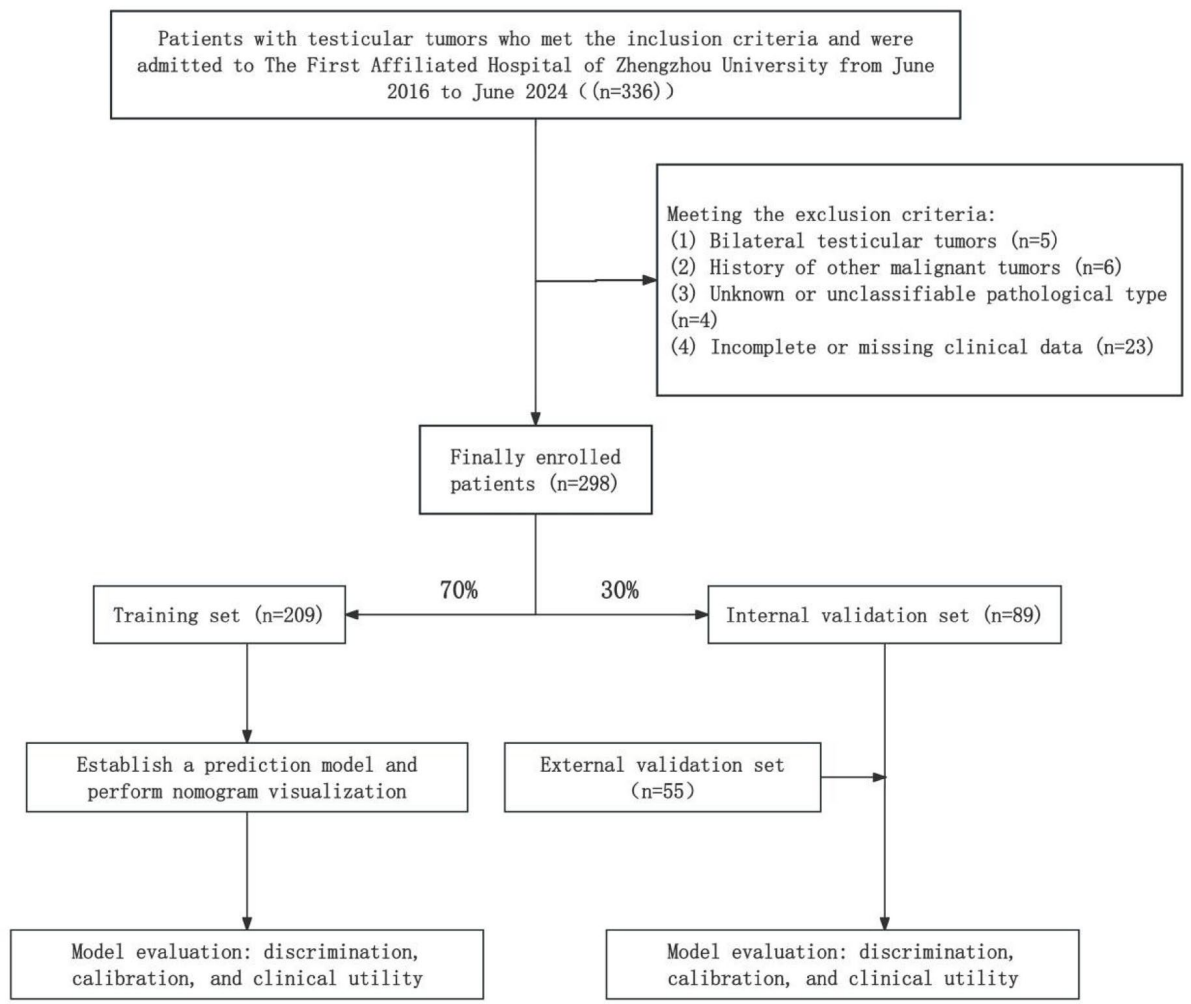
Research protocol diagram.

This retrospective study enrolled patients diagnosed with testicular tumors from the First Affiliated Hospital of Zhengzhou University between June 2016 and June 2024. A total of 298 patients who met the inclusion and exclusion criteria were included. Using a computer-generated random number sequence, the patients were allocated into a training set (209 patients, 70%) and an internal validation set (89 patients, 30%). In addition, 55 patients from two other tertiary hospitals in Henan Province, treated between January 2019 and January 2024 and meeting the same criteria, were included as an external validation set. The training cohort was used to develop the prediction model, which was subsequently evaluated using both internal and external validation sets(Fig. 1).

**Inclusion criteria**:

1. Pathologically confirmed primary testicular tumor
2. Underwent radical orchiectomy or testis-sparing surgery

**Exclusion criteria**:

1. Bilateral testicular tumors
2. History of other malignant tumors
3. Unknown or unclassifiable pathological type
4. Incomplete or missing clinical data

The study was approved by the Ethics Committee of the First Affiliated Hospital of Zhengzhou University (Approval No: 2024-KY-1993). Due to the retrospective nature of this study, the requirement for informed consent was waived. All data were anonymized to protect patient privacy, and the study was conducted in accordance with the ethical principles of the Declaration of Helsinki (2024 revision).

For the binary logistic regression prediction model, sample size estimation was performed using the widely accepted 10 Events Per Variable (10 EPV) rule^5^. Based on preliminary analyses, the final model was anticipated to include 3–5 predictor variables. Accounting for an estimated 20% data attrition rate, the minimum required sample size was calculated to be between 120 and 200 cases using Formula (2.1)^6^.

2.1 $$\:Sample\:Size = \frac{{Number\:of\:\:Variables \times EPV}}{{1 - Incidence\:Rate}}$$

### Data collection and definitions

Clinical data were retrieved from the hospital’s electronic medical record system. The following variables were collected and defined

#### Age, testicular mass, and cryptorchidism history

Age was defined as the patient’s age at the initial diagnosis. Information regarding testicular masses and a history of cryptorchidism was obtained by reviewing inpatient medical records.

#### Pathological diagnosis

Pathological results were based on standard pathological reports. Each routine pathological section was initially evaluated by one pathologist and subsequently reviewed by a senior pathologist. Comprehensive pathological diagnosis was established according to the 4th^7^ and 5th^8^ editions of the WHO classification of testicular tumors, incorporating immunohistochemical findings. In cases of diagnostic disagreement between the two pathologists, the opinion of the senior pathologist prevailed, or a consensus was reached through consultation or a pathology review meeting. The pathological diagnosis served as the gold standard for categorizing patients into malignant and benign tumor groups.

#### Ultrasonic examination

Ultrasound examinations were performed using the following devices: Fuji Film 850 (Japan; probe frequency 2–12 MHz), Philips EPIQ 7 C (Netherlands; probe frequency 4–18 MHz), and GE LOGIQ C9 (USA; probe frequency 9–12 MHz).We had the same dedicated genitourinary ultrasound physician perform scans on 61 consecutive patients who presented for genitourinary ultrasound examinations using three ultrasound machines (Fuji, Philips, and GE). The results yielded a Kappa value of 0.6051, indicating good consistency.

Tumor Laterality and Size: Tumor laterality was recorded as left or right. Tumor size was defined as the maximum diameter (cm) measured in the largest cross-sectional plane.

Boundary: A clear border was defined as smooth, sharp, and well-demarcated from surrounding tissues. An unclear border referred to an irregular, blurred, or spiculated margin with adhesion or indistinct boundaries.

Echogenicity: Based on reflected ultrasound signals, echogenicity was classified as hypoechoic, hyperechoic, or mixed.

CDFI: Color Doppler flow imaging (CDFI) was used to assess the distribution of blood flow within the mass. Blood flow signals were graded according to the Adler semi-quantitative grading system^9^: Grade 0, no blood flow; Grade I, 1–2 punctate flow signals; Grade II, ≥ 3 punctate signals or one linear vessel; Grade III, multiple linear or branching vessels. To mitigate potential data imbalance due to multi-category classification, Grades 0 and I were combined and defined as “Grade 0–I”.

#### Serum tumor markers

Serum levels of alpha-fetoprotein (AFP), human chorionic gonadotropin (hCG), and lactate dehydrogenase (LDH) were measured. All samples were analyzed in the central laboratory: AFP and hCG were measured using a Roche cobas e801 electrochemiluminescence immunoassay analyzer, with normal ranges of 0–10 ng/mL and 0–5 ng/mL, respectively. LDH was measured using a Roche cobas 8000 automated biochemical analyzer, with a normal range of 75–245 U/L.

All study variables were obtained prior to surgery. Pathological diagnosis was used as the outcome variable.

All clinical variables from the training, internal validation, and external validation cohorts were initially compared using SPSS (version 25.0). Univariate logistic regression was first performed on the training cohort to assess the association between each variable and tumor type. To avoid omitting potentially important predictors, prevent overfitting, and enhance model generalizability, all variables were subsequently included in a Least Absolute Shrinkage and Selection Operator (LASSO) regression analysis. By applying L1 regularization, LASSO identified variables with non-zero coefficients. Those selected variables were then tested for multicollinearity. Only variables that passed the multicollinearity diagnostic were entered into the multivariate logistic regression analysis to identify independent discriminative factors for distinguishing between benign and malignant testicular tumors.

The independent discriminative factors identified from the multivariate logistic regression were incorporated into the nomogram prediction model. The ‘rms’ package in R software was used to visualize the model. The model’s discriminative ability was assessed by plotting the receiver operating characteristic (ROC) curve and calculating the area under the curve (AUC). Calibration was evaluated using calibration plots, the Spiegelhalter Z-test, and the Brier score. Clinical utility was examined via decision curve analysis (DCA)^10^. The nomogram was subsequently validated in both the internal and external validation cohorts using ROC curves, calibration curves, and DCA. Finally, the model was deployed as a dynamic nomogram on a personal website to facilitate convenient online access and use.

All analyses were conducted using R software (version 4.4.2; www.r-project.org) and SPSS (version 25.0). Continuous variables with non-normal distributions are presented as median and interquartile range (IQR) [M (Q1, Q3)], and group comparisons were performed using the Kruskal-Wallis test. Categorical data are expressed as frequency (percentage), and comparisons between groups were made using the Chi-square test or Fisher’s exact test, as appropriate. The Mann-Whitney U test was used for ordinal data. Independent discriminative factors for benign and malignant testicular tumors were identified in the training cohort using LASSO regression followed by multivariate logistic regression. The final set of selected predictors was used to refit the model and construct the nomogram. The nomogram’s performance was evaluated using ROC curves, calibration curves, and DCA, and subsequently validated in the validation cohorts. A two-tailed P-value < 0.05 was considered statistically significant.

## Result

**Fig. 2:**
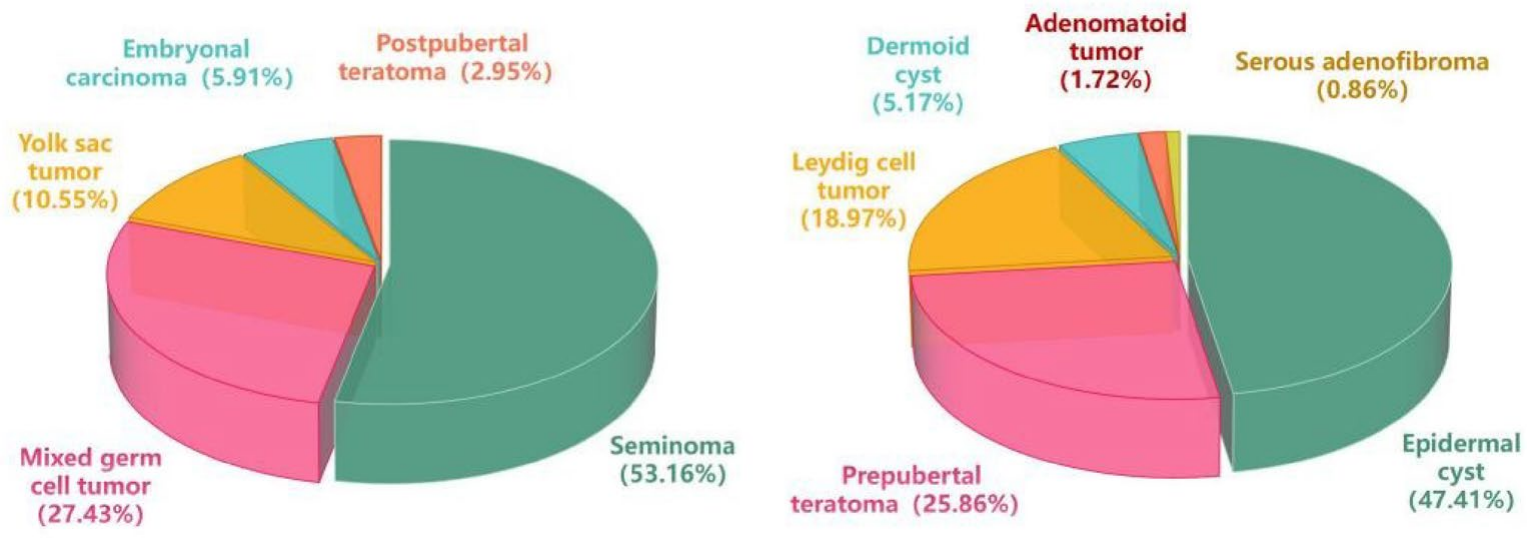
Pathological distribution of enrolled patients.

A total of 353 patients were included in this study. Among them, 237 cases (67.14%) were diagnosed with malignant testicular tumors. The most common malignant subtype was seminoma (126 cases, 53.16%).It should be noted that the 2016 WHO classification of testicular tumors reclassified teratomas into two distinct categories based on their origin relative to germ cell neoplasia in situ (GCNIS): prepubertal(GCNIS-unrelated) and postpubertal(GCNIS-derived)^7^. Somatic malignant components are exclusively observed in postpubertal teratoma, which carries a potential for recurrence or metastasis, with approximately 22–37% of patients developing lymph node metastasis.Benign testicular tumors were identified in 116 patients (32.86%). The most frequent benign pathology was epidermoid cyst (55 cases, 47.41%), while the least common was serous adenofibroma (1 case, 0.86%).(Fig. 2).

### Comparison of clinical characteristics across training, internal validation, and external validation sets

Clinical characteristics were compared among the training, internal validation, and external validation cohorts. The results showed no statistically significant differences (*P* > 0.05) in age, tumor size, tumor border, testicular microlithiasis, AFP, hCG, LDH, tumor laterality, testicular mass, history of cryptorchidism, internal echogenicity, or internal blood flow. These findings indicate that the three datasets were well-balanced and comparable(Table 1).

**Table 1:** Comparison of clinical data between the training set and validation sets.

	n（%）or M（Q1，Q3）				*c* *²/H*	*P*
	Total（n=353）	Training set（n=209）	Internal validation set（n=89）	External validation set（n=55）		
Age	27.00（16.00，35.00）	26.00（16.00，34.00）	27.00（11.00，34.00）	30.00（19.50，41.50）	4.98	0.083
Tumor size	3.00（1.90，4.50）	3.00（2.00，4.50）	3.30（1.90，5.00）	2.60（1.85，3.60）	2.92	0.232
Boundary					0.27	0.874
Clear	226（64.02）	132（63.16）	59（66.29）	35（63.64）		
Unclear	127（35.98）	77（36.84）	30（33.71）	20（36.36）		
Testicular microlithiasis					1.31	0.518
No	306（86.69）	184（88.04）	74（83.15）	48（87.27）		
Yes	47（13.31）	25（11.96）	15（16.85）	7（12.73）		
AFP					5.27	0.072
Nomal	250（70.82）	142（67.94）	62（69.66）	46（83.64）		
Increase	103（29.18）	67（32.06）	27（30.34）	9（16.36）		
hCG					0.30	0.862
Nomal	247（69.97）	144（68.90）	64（71.91）	39（70.91）		
Increase	106（30.03）	65（31.10）	25（28.09）	16（29.09）		
LDH					3.34	0.188
Nomal	226（64.02）	128（61.24）	57（64.04）	41（74.55）		
Increase	127（35.98）	81（38.76）	32（35.96）	14（25.45）		
Laterality					3.30	0.192
Left	157（44.48）	101（48.33）	36（40.45）	20（36.36）		
Right	196（55.52）	108（51.67）	53（59.55）	35（63.64）		
Testicular Pain and Swelling					1.35	0.509
No	253（71.67）	151（72.25）	66（74.16）	36（65.45）		
Yes	100（28.33）	58（27.75）	23（25.84）	19（34.55）		
Cryptorchidism					5.13	0.077
No	312（88.39）	179（85.65）	80（89.89）	53（96.36）		
Yes	41（11.61）	30（14.35）	9（10.11）	2（3.64）		
Echogenicity					2.26	0.689
Hyperechoic	25（7.08）	14（6.70）	7（7.87）	4（7.27）		
Hypoechoic	209（59.21）	120（57.42）	52（58.43）	37（67.27）		
Mixed	119（33.71）	75（35.89）	30（33.71）	14（25.45）		
CDFI					0.33	0.847
0-I	187（52.97）	109（52.15）	47（52.81）	31（56.36）		
Ⅱ	74（20.96）	51（24.40）	14（15.73）	9（16.36）		
Ⅲ	92（26.06）	49（23.44）	28（31.46）	15（27.27）		

### Analysis of discriminative factors for benign and malignant testicular tumors in the training set

The training set consisted of 209 patients, including 144 (68.90%) with malignant tumors. Univariate logistic regression identified the following factors as statistically significant discriminators between benign and malignant testicular tumors: age (OR = 1.05, 95% CI: 1.02–1.07, *P* < 0.001), tumor size (OR = 3.47, 95% CI: 2.41–5.00, *P* < 0.001), boundary (OR = 2.83, 95% CI: 1.44–5.58, *P* = 0.003), AFP (OR = 5.92, 95% CI: 2.53–13.86, *P* < 0.001), hCG (OR = 11.21, 95% CI: 3.87–32.49, *P* < 0.001), LDH (OR = 2.03, 95% CI: 1.08–3.83, *P* = 0.029), testicular pain and swelling (OR = 2.07, 95% CI: 1.01–4.25, *P* = 0.047), cryptorchidism (OR = 3.36, 95% CI: 1.12–10.07, *P* = 0.030), and CDFI (Grade II: OR = 4.26, 95% CI: 1.89–9.59, *P* < 0.001; Grade III: OR = 10.26, 95% CI: 3.45–30.51, *P* < 0.001).

In contrast, testicular microlithiasis, laterality, and echogenicity showed no statistically significant association (*P* > 0.05) with tumor malignancy(Table 2).

**Table 2:** Univariate logistic regression.

Age	β	S.E	Z	OR(95CI%)	*P*
	0.05	0.01	3.98	1.05(1.02–1.07)	< 0.001
Tumor size	1.24	0.19	6.66	3.47(2.41–5.00)	< 0.001
Boundary					
Clear					
Unclear	1.04	0.35	3.02	2.83(1.44–5.58)	0.003
Testicular Microlithiasis					
No					
Yes	−0.25	0.45	−0.56	0.78(0.32–1.87)	0.573
AFP					
Nomal					
Increase	1.78	0.43	4.09	5.92(2.53–13.86)	< 0.001
hCG					
Nomal					
Increase	2.42	0.54	4.45	11.21(3.87–32.49)	< 0.001
LDH					
Nomal					
Increase	0.71	0.32	2.19	2.03(1.08–3.83)	0.029
Laterality					
Left					
Right	0.14	0.30	0.47	1.15(0.64–2.07)	0.635
Testicular pain and swelling					
No					
Yes	0.73	0.37	1.99	2.07(1.01–4.25)	0.047
Cryptorchidism					
No					
Yes	1.21	0.56	2.17	3.36(1.12–10.07)	0.030
Echogenicity					
Hyperechoic					
Hypoechoic	1.01	0.57	1.77	2.75(0.89–8.45)	0.077
Mixed	0.63	0.59	1.08	1.88(0.60–5.96)	0.280
CDFI					
0-I					
Ⅱ	1.45	0.41	3.50	4.26(1.89–9.59)	< 0.001
Ⅲ	2.33	0.56	4.19	10.26(3.45–30.51)	< 0.001

**Fig. 3:**
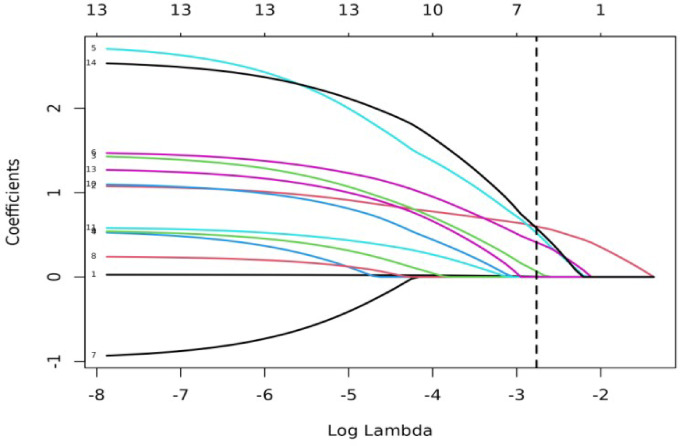
Coefficient path plot for LASSO regression.

**Fig. 4:**
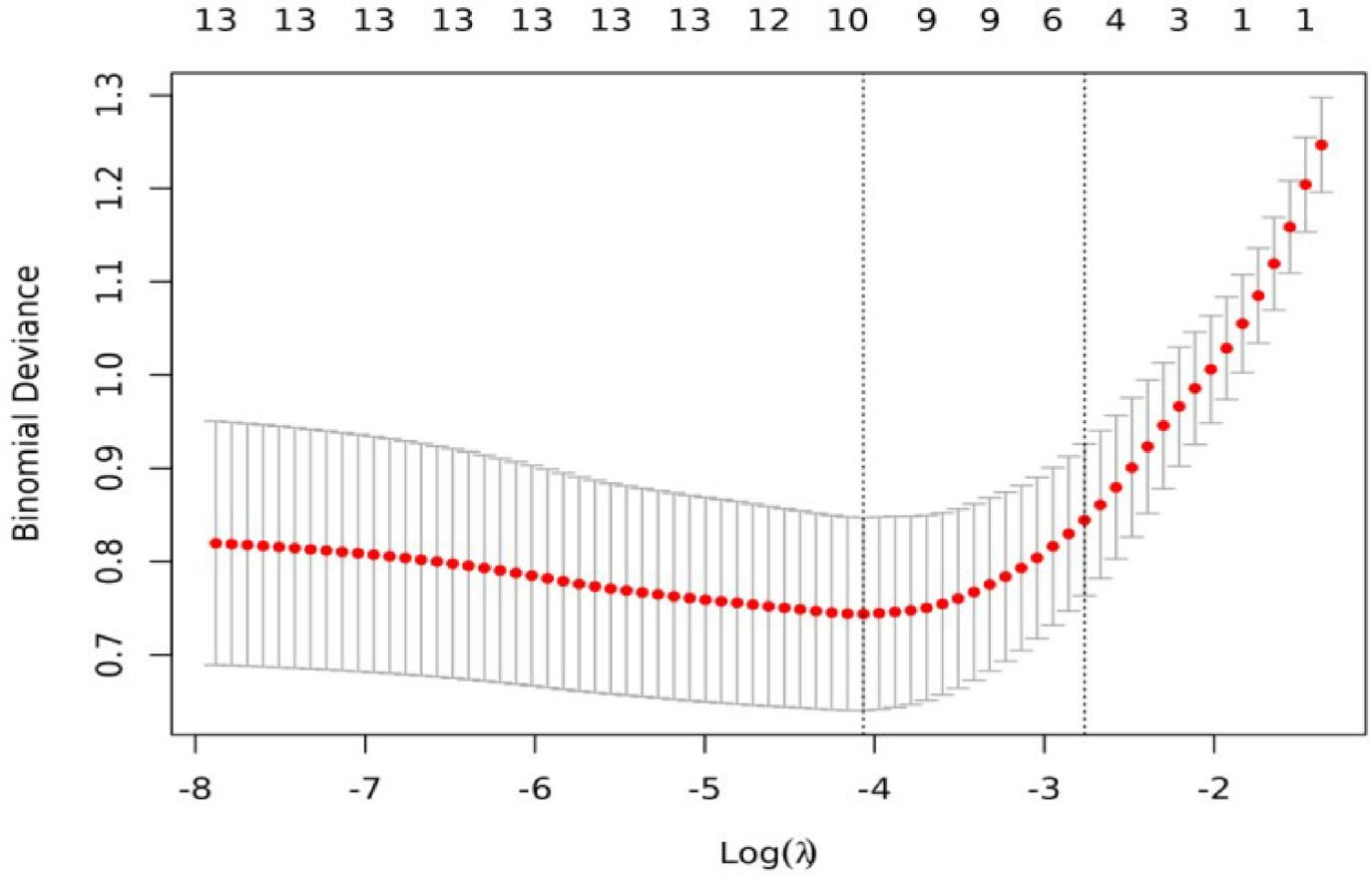
Cross-validation plot for LASSO regression.

To avoid losing important variables, all variables were subjected to LASSO regression.To select a model with good performance and relative simplicity, six non-zero coefficient variables were identified: tumor size, CDFI, AFP, hCG, Boundary, and age(Figs. 3 and 4).

Results showed that the non-zero coefficient variables, in descending order of their coefficients, were: tumor size, grade III CDFI, increased AFP, increased hCG, unclear boundary, and age(Table 3).

**Table 3:** Regression coefficient.

(Intercept)	Coefficient
	−1.51176124
Tumor size	0.59900354
Grade III CDFI	0.57725034
Increased AFP	0.52065652
Increased hCG	0.41062753
Unclear boundary	0.06782327
Age	0.00412988

**Fig. 5:**
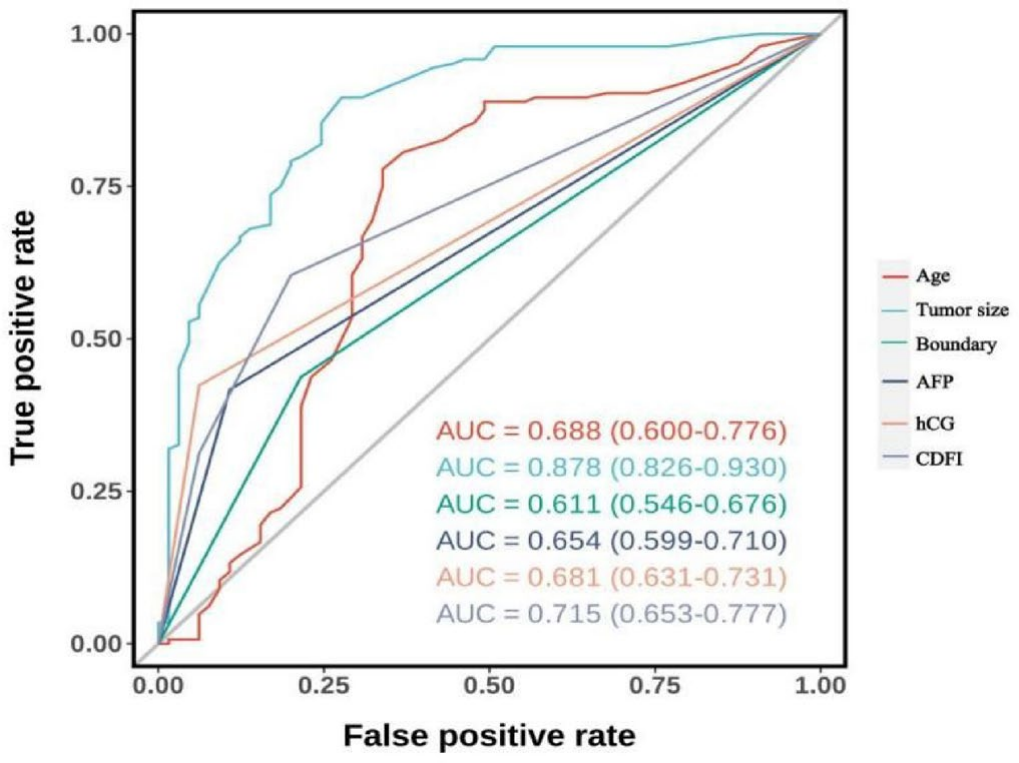
ROC curves for each single variable in logistic regression.

Subsequent univariate logistic regression analysis of these six non-zero coefficient variables and evaluation of their individual AUC values via ROC curves revealed that, with the exception of tumor size which demonstrated a relatively high AUC (AUC = 0.878, 95% CI: 0.826–0.930), the other five indicators showed comparatively lower discriminatory power. These findings indicate that relying on any single indicator for differentiating benign from malignant testicular tumors is unreliable(Fig. 5).

The six non-zero coefficient variables that passed the collinearity diagnostic were included in the multivariate logistic regression model. Final analysis identified five independent discriminative factors for distinguishing benign from malignant testicular tumors: tumor size (OR = 2.70, 95% CI: 1.80–4.05, *P* < 0.001), Boundary(OR = 3.99, 95% CI: 1.34–11.86, *P* = 0.013), AFP (OR = 7.49, 95% CI: 1.92–29.25, *P* = 0.004), hCG (OR = 5.21, 95% CI: 1.30–20.95.30.95, *P* = 0.020), and CDFI (Grade II: OR = 4.23, 95% CI: 1.35–13.26, *P* = 0.014; Grade III: OR = 16.26, 95% CI: 3.68–71.83, *P* < 0.001).(Table 4).

**Table 4:** Multivariate logistic regression.

Age	β	S.E	Z	OR(95CI%)	*P*
	0.03	0.02	1.89	1.03(1.00–1.07.00.07)	0.059
Tumor size	0.99	0.21	4.81	2.70(1.80–4.05)	< 0.001
Boundary					
Clear					
Unclear	1.38	0.56	2.49	3.99(1.34–11.86)	0.013
AFP					
Nomal					
Increase	2.01	0.70	2.90	7.49(1.92–29.25)	0.004
hCG					
Nomal					
Increase	1.65	0.71	2.32	5.21(1.30–20.95.30.95)	0.020
CDFI					
0-I					
Ⅱ	1.44	0.58	2.47	4.23(1.35–13.26)	0.014
Ⅲ	2.79	0.76	3.68	16.26(3.68–71.83)	< 0.001

### Development of a nomogram prediction model for differentiating benign and malignant testicular tumors

**Fig. 6:**
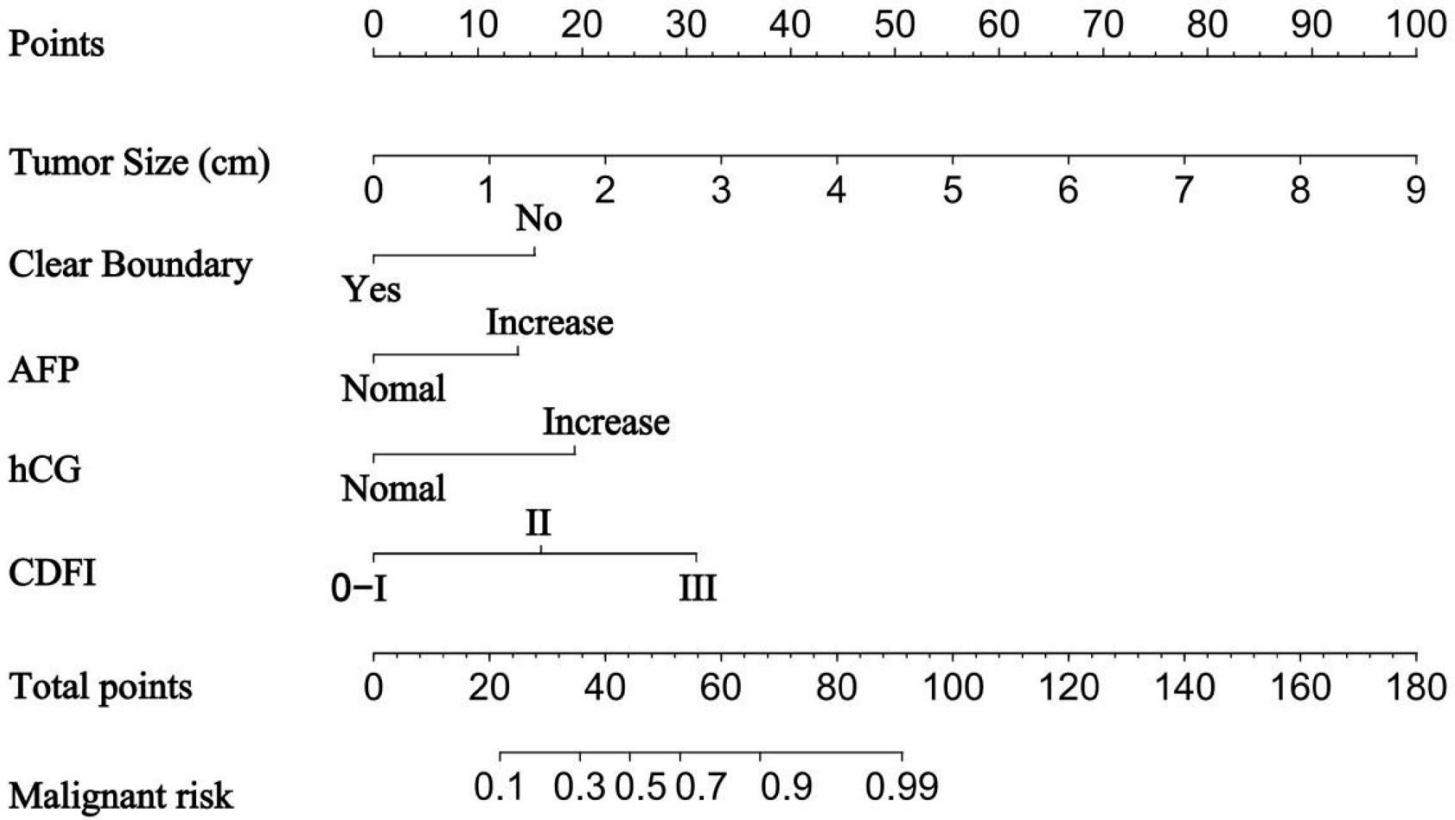
Nomogram.

Through LASSO regression and multivariate logistic regression analysis, five variables were identified as independent discriminative factors for distinguishing between benign and malignant testicular tumors: tumor size, boundary, AFP, hCG, and CDFI. Based on these factors, a visual nomogram was constructed using the rms package in R software**(**Fig. 6**)**. To facilitate clinical application, the model was deployed online as a dynamic nomogram accessible at https://nomogram98.shinyapps.io/dynnomapp/. This tool allows clinicians to input patient-specific data and obtain individualized risk estimates, thereby supporting the development of personalized treatment plans.

**Fig. 7:**
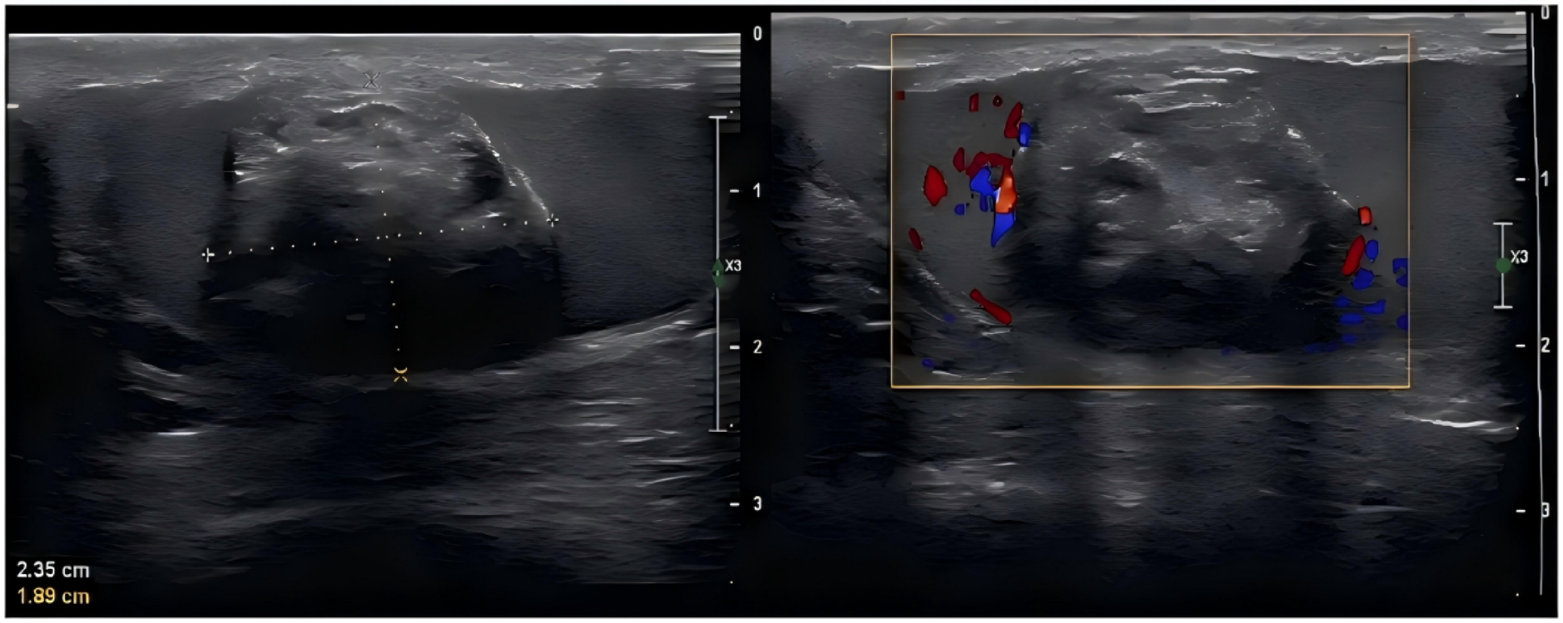
A scale bar is shown on the right side of the image.The transverse diameter (white dashed line) of the patient’s tumor is 2.35 cm, and the longitudinal diameter (yellow dashed line) is 1.89 cm.

**Fig. 8:**
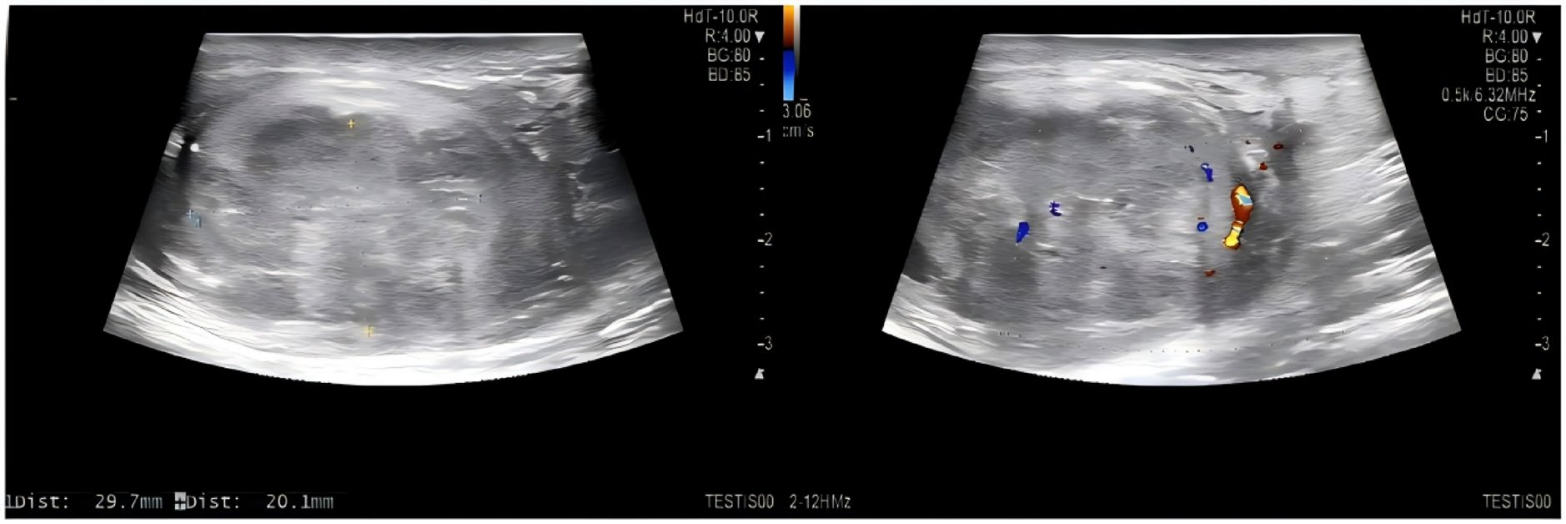
The transverse diameter (blue dashed line) of the patient’s tumor is 2.97 cm, and the longitudinal diameter (yellow dashed line) is 2.01 cm.

Supplementary Figure details the application scenarios, workflow, and clinical performance of the nomogram(Fig. 7&8)

### Evaluation of the nomogram prediction model

**Fig. 9:**
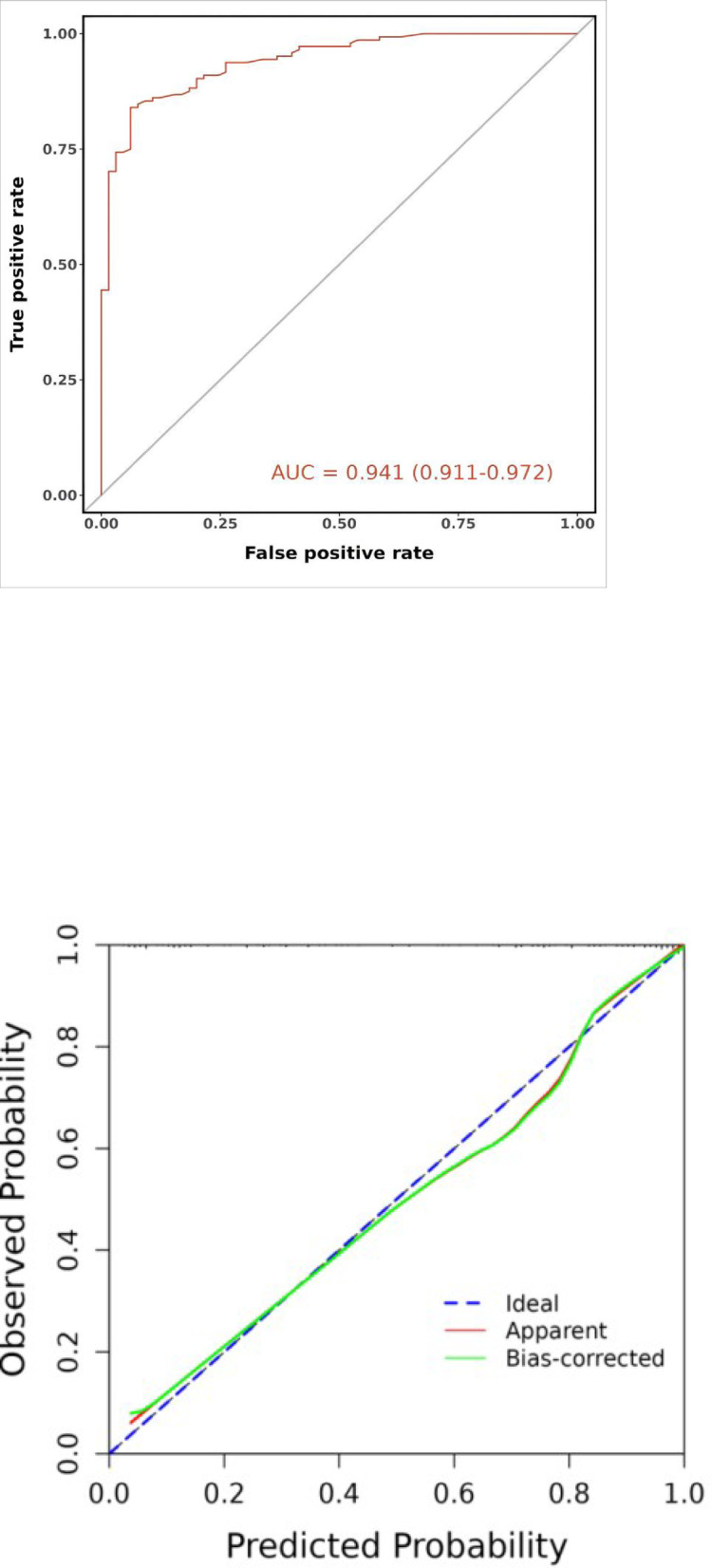

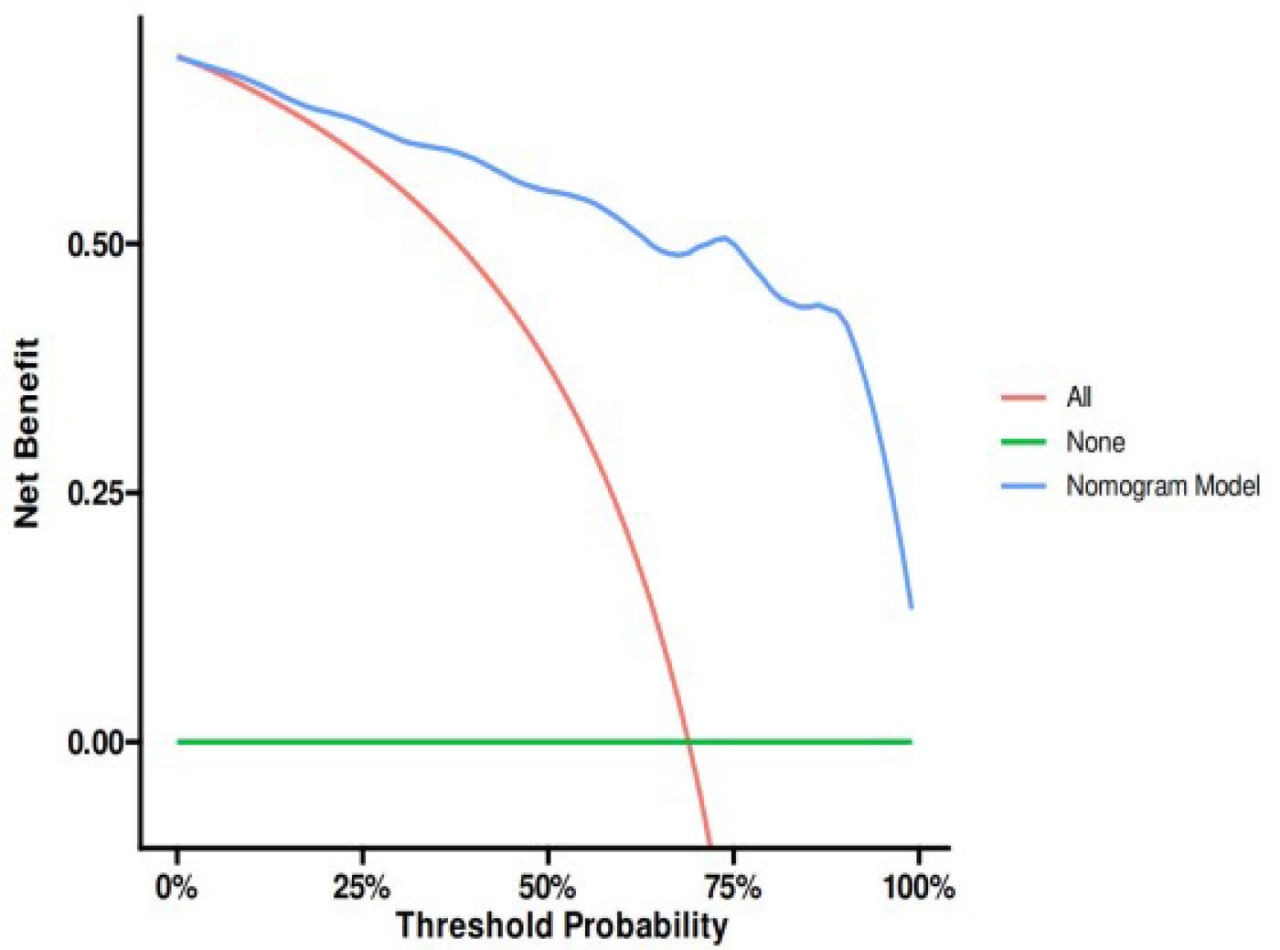
Training set’s ROC curve (**A**), calibration curve(**B**) and decision curve analysis curve(**C**).

The nomogram prediction model demonstrated an AUC of 0.941 (95% CI: 0.911–0.972). Accuracy was 0.871 (95% CI: 0.818–0.913), with a sensitivity of 0.938 (95% CI: 0.880–0.997) and specificity of 0.840 (95% CI: 0.780–0.900). The positive predictive value was 0.726 (95% CI: 0.631–0.822), and the negative predictive value was 0.968 (95% CI: 0.937–0.999). The optimal cutoff value was determined to be 0.744. The Spiegelhalter Z-test yielded a P-value of 0.827, and the Brier score was 0.093. Furthermore, decision curve analysis (DCA) indicated that the nomogram provided higher net benefit than the reference strategies across a threshold probability range of approximately 2% to 100%. These results collectively demonstrate that the nomogram prediction model exhibits strong discriminatory power, good calibration, and favorable clinical utility**(**Fig. 9**)**.

### Validation of the nomogram prediction model

**Fig. 10:**
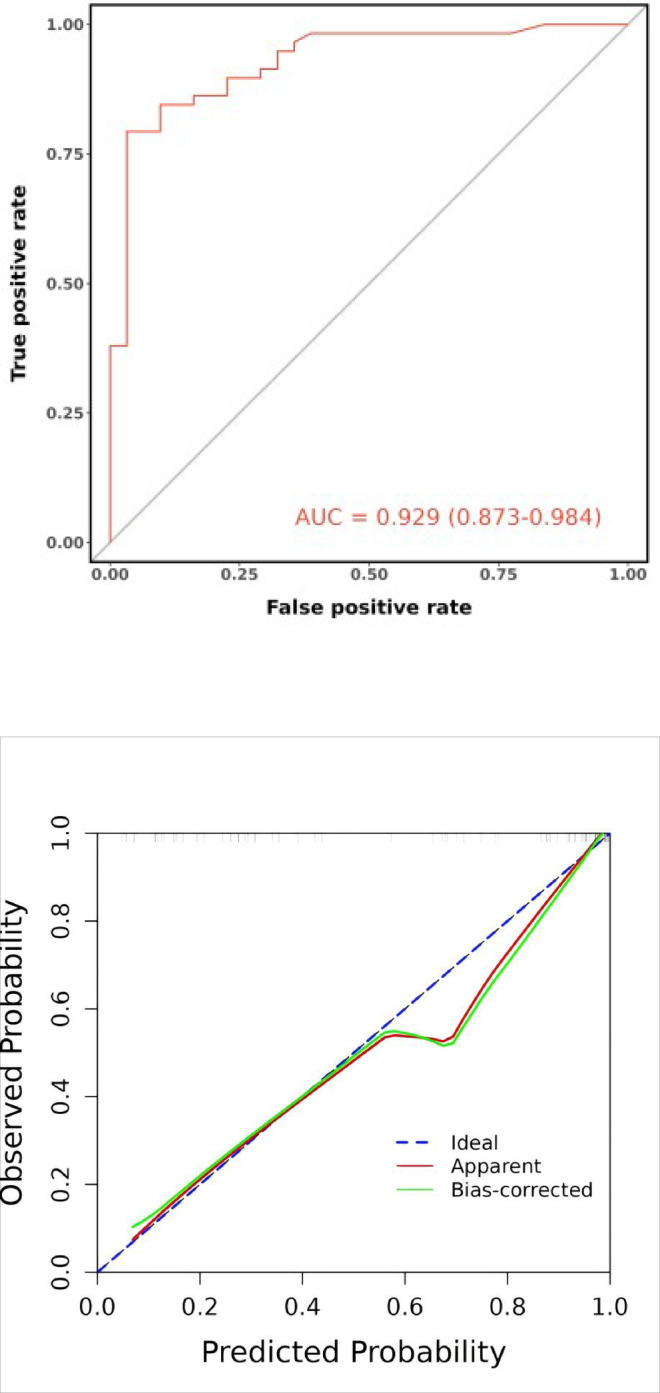

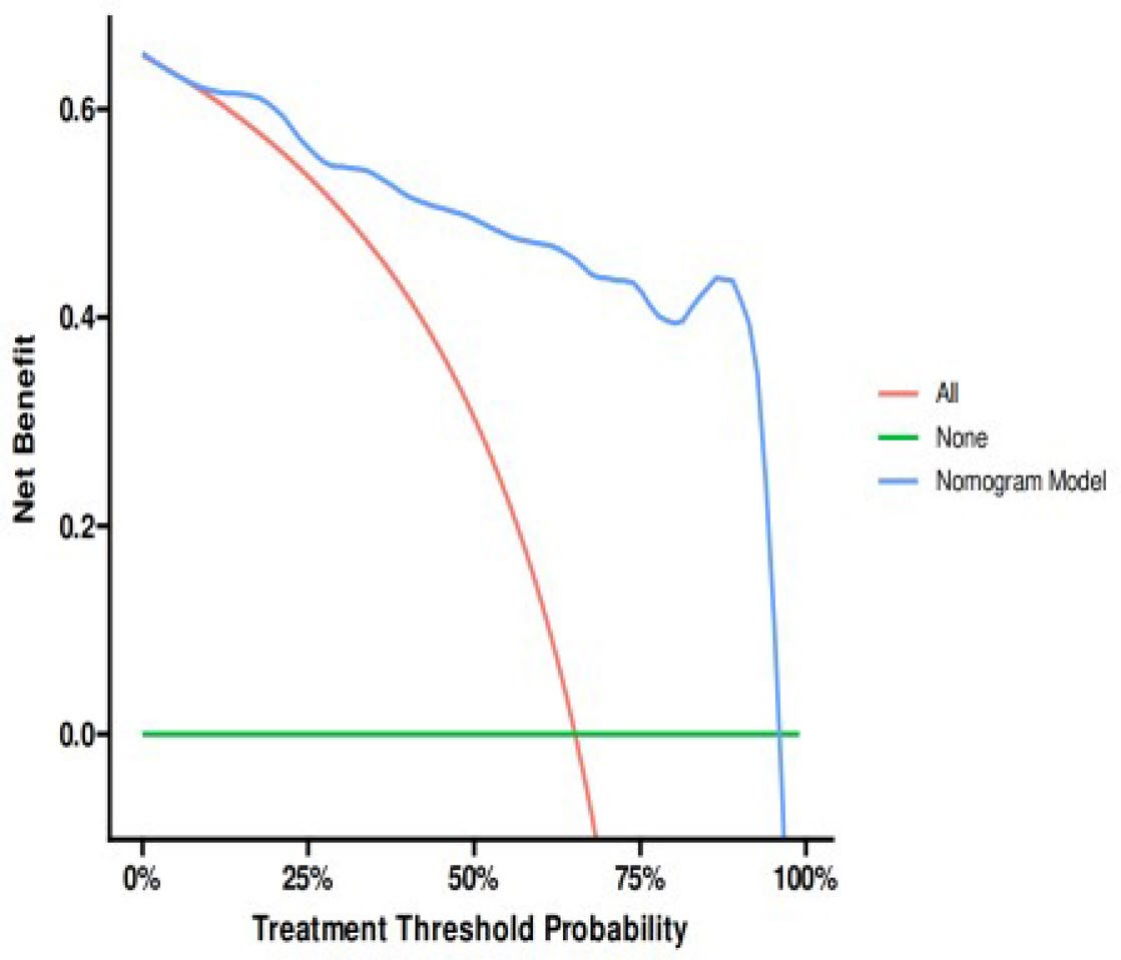
Internal validation set’s ROC curve (**A**), calibration curve(**B**) and decision curve analysis curve(**C**).

In the internal validation set, the nomogram demonstrated an AUC of 0.929 (95% CI: 0.873–0.984), with an accuracy of 0.854 (95% CI: 0.763–0.920). Sensitivity was 0.903 (95% CI: 0.799–1.000) and specificity was 0.828 (95% CI: 0.730–0.925). The positive predictive value was 0.737 (95% CI: 0.597–0.877), and the negative predictive value was 0.941 (95% CI: 0.877–1.000). The optimal cutoff value remained at 0.744. The Spiegelhalter Z-test yielded a P-value of 0.080, and the Brier score was 0.105. Decision curve analysis showed that the nomogram’s DCA curve demonstrated superior net benefit compared to the reference lines across a threshold probability range of 10% to 95%(Fig. 10).

**Fig. 11:**
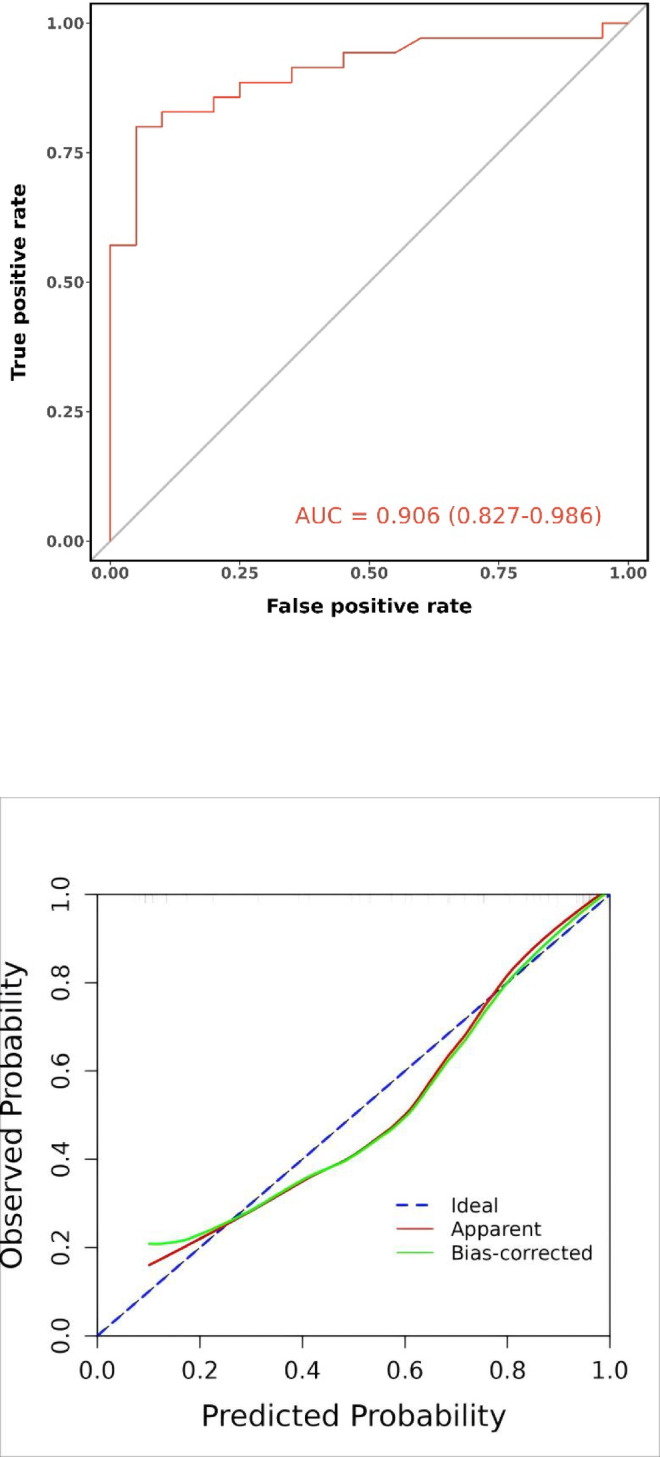

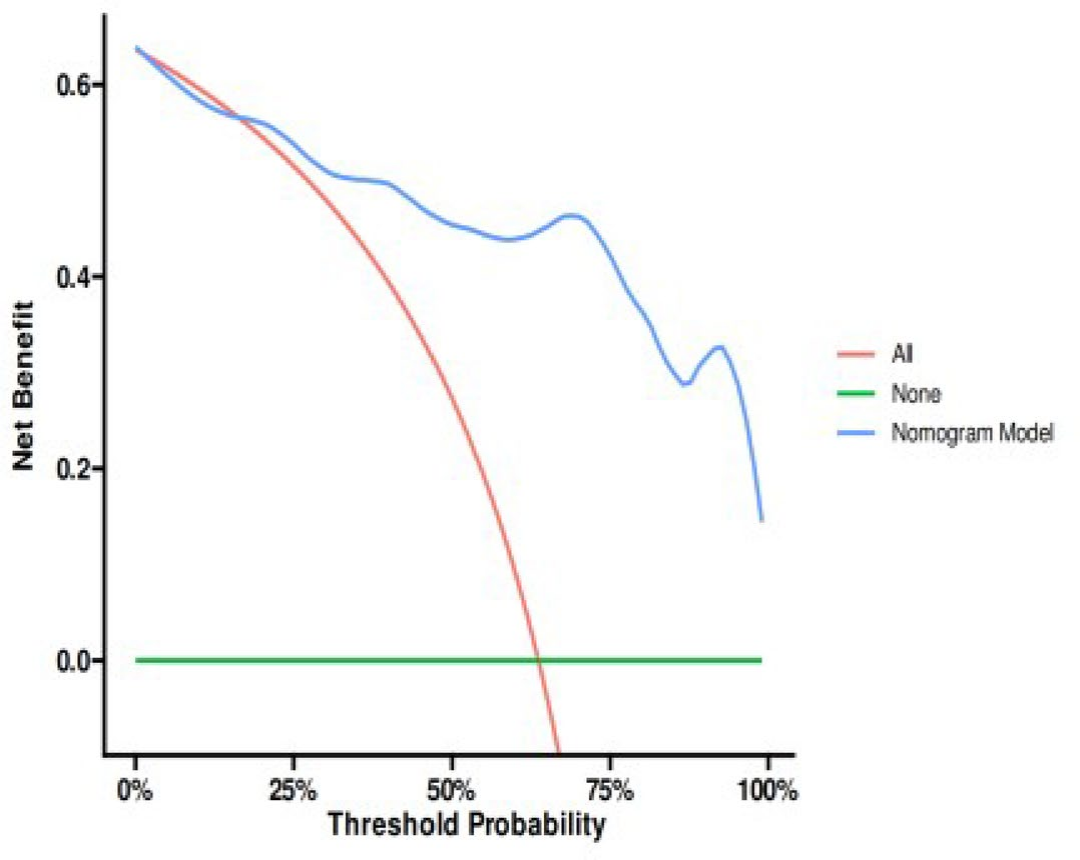
External validation set’s ROC curve (**A**), calibration curve(**B**) and decision curve analysis curve(**C**).

In the external validation set, the model achieved an AUC of 0.906 (95% CI: 0.827–0.986), with an accuracy of 0.836 (95% CI: 0.712–0.922). Sensitivity reached 0.950 (95% CI: 0.854–1.000), while specificity was 0.771 (95% CI: 0.632–0.911). The positive predictive value was 0.704 (95% CI: 0.531–0.876), and the negative predictive value was 0.964 (95% CI: 0.896–1.000). The optimal cutoff value was consistent at 0.744. The Spiegelhalter Z-test result was 0.497, with a Brier score of 0.120. The DCA curve indicated enhanced clinical utility within the threshold probability range of 15% to 100%**(**Fig. 11**)**.

Both internal and external validation results confirm that our model maintains excellent predictive performance, demonstrating robust generalizability and clinical applicability.

We determined the optimal cutoff value of the nomogram using the Youden index, which was 0.744 (corresponding to 55 points).Patients with a score > 55 were classified as the high-risk group and recommended for radical orchiectomy.However, for patients with a score below 55 points, direct testis-sparing surgery carries a relatively high risk.We further divided patients with a score ≤ 55 into two subgroups.

The overall risk stratification of the nomogram is as follows:

Low-risk (≤ 44 points): testis-sparing surgery.

Intermediate-risk (44–55 points): intraoperative frozen section.

High-risk group(> 55 points): radical orchiectomy.

## Discussion

The early differentiation between benign and malignant testicular tumors remains a significant clinical concern, particularly for young men with fertility preservation needs. Accurate preoperative identification of tumor nature can help avoid unnecessary radical orchiectomy (RO), thereby preserving endocrine function, maintaining testicular integrity, and reducing risks of late-onset hypogonadism and psychological sequelae. For patients identified by the nomogram as having low malignant risk, testis-sparing surgery (TSS) or active surveillance may be prioritized after thorough communication with patients and their families.

The development of nomogram models for testicular tumors is still in the initial stage, especially for rare pathological subtypes such as yolk sac tumor^11^ and lymphoma^12^. Previous studies have mainly focused on the prognosis of patients with testicular tumors^13^, while neglecting the significance of tumor nature in guiding the selection of treatment strategies.Song et al.^14^ developed a nomogram incorporating seven parameters including age, tumor size, cryptorchidism, echogenicity, CDFI, AFP, and hCG, ultimately identifying four independent discriminative factors: tumor size, echogenicity, hCG, and CDFI, achieving an AUC of 0.92. Fang et al.^15^ combined machine learning with radiomics, establishing a clinical-deep learning-radiomics model using ultrasound images and clinical data from training (*n* = 158), validation (*n* = 68), and external validation (*n* = 49) cohorts, which demonstrated excellent performance with AUCs of 0.851 and 0.834 in internal and external validation, respectively. Addressing limitations of previous studies such as small sample sizes, incomplete validation, and limited parameters, our study developed and optimized a nomogram prediction model with comprehensive internal and external validation.

Demographic factors show significant correlations with testicular tumor differentiation and pathological distribution.While LASSO and univariate regression identified age as a discriminative factor, its individual AUC was only 0.688, showing limited discriminatory power. Age was not retained in the multivariate model, likely attributable to overlapping age distributions between benign and malignant cases in our sample. In our entire study cohort, patients with epidermoid cysts were generally younger; notably, however, non-seminomatous tumor patients in the malignant group also occurred predominantly in young adults (with a median age of 23 years), resulting in a certain age overlap between the two groups. This may cause considerable confusion and potential misguidance for clinicians. Although age was not incorporated into the final model, careful preoperative differentiation remains crucial for pediatric and infant patients given their immature gonadal development.

Imaging examination serves as a crucial modality for differentiating between benign and malignant testicular tumors. Numerous studies have indicated that tumor size can be a discriminative factor^16,17^. Larger testicular tumors are more likely to be malignant^18^. Konstantatou et al.^19^, in a study comparing 31 malignant and 17 benign testicular tumors, found significant correlations between malignancy and both tumor size (*P* = 0.001) and abundant blood flow (*P* < 0.001). In our study, tumor size was represented by the maximum diameter measured across different ultrasound sections. The median size was 3.90 cm for malignant tumors and 1.60 cm for benign tumors. Statistical analysis in the training cohort confirmed tumor size as an independent discriminative factor (OR = 2.70, 95% CI: 1.80–4.05, *P* < 0.001). Tumor size carried substantial weight in our model, and the wide size range included (up to nearly 9 cm) enhances the model’s reliability and practical applicability for encompassing most clinical scenarios. Analysis revealed that tumor size alone achieved an AUC > 0.8, and its inclusion significantly improved the model’s discriminatory power and predictive performance.

Beyond tumor size, other sonographic features such as boundary, echogenicity, and CDFI also exhibit differences between benign and malignant lesions. Some studies suggest that most histologically malignant testicular masses present as distinctly hypoechoic on preoperative ultrasound^20^. Pozza et al.^21^ reported a strong association between hypoechogenicity and malignancy (OR = 11.509, *P* = 0.036). However, findings from Huang et al.^22^ differed, showing no significant differences between benign and malignant tumors regarding hypoechoic appearance, calcification, irregular margins, or presence of testicular microlithiasis. The definitive diagnostic value of boundary, echogenicity, and testicular microlithiasis for differentiation remains inconclusive. While testicular microlithiasis indicates testicular atrophy and degeneration, its evidence as a reliable discriminative factor is still insufficient. Patients with testicular microlithiasis should maintain vigilance with increased follow-up frequency for early detection of potential malignancies.

As testicular tumors enlarge, malignant ones may undergo degenerative necrosis. Disorganized internal architecture can sonographically manifest as ill-defined borders and heterogeneous echogenicity, while simultaneously stimulating neoangiogenesis leading to abundant internal flow. In contrast, benign tumors typically grow slower, expand progressively, and maintain relatively uniform structure, often appearing as well-circumscribed, homogeneous masses on ultrasound. Sonographic features also correlate closely with histopathological subtypes. For instance, most seminomas present as well-defined, hypoechoic masses with rich vascularity^23^, although internal fibrous septa can create hyperechoic strands, resulting in a heterogeneous “map-like” pattern. Mixed malignant germ cell tumors, being highly aggressive and histologically diverse, frequently undergo hemorrhage, necrosis, and cystic change, often appearing as mixed-echoic or hypoechoic complex masses. Epidermoid cysts typically present as well-circumscribed cystic lesions with scant blood flow, most commonly demonstrating “onion-skin” or “target” signs sonographically^24^. The former results from layered keratinized debris from desquamated squamous epithelial cells, while the latter features a predominantly hypoechoic center with a peripheral hyperechoic rim. Embryonal carcinoma, a highly heterogeneous tumor, exhibits rapid growth, strong invasiveness, lacks a true capsule, and often demonstrates indistinct borders from surrounding tissues.The differentiation of testicular tumours with US continues to be challenging^25^.

Serum tumor markers are indispensable in the diagnosis and management of testicular tumors. AFP, normally present in fetal serum, is produced by the yolk sac and later by the fetal liver. As secretion continues postnatally, elevated AFP levels occur not only in hepatic and testicular tumors but also normally during infancy and early childhood. Typically, AFP is elevated in nearly all yolk sac tumors or mixed germ cell tumors containing yolk sac elements, as these cells differentiate into primitive endodermal cells capable of secreting AFP. In contrast, AFP levels are generally not elevated in pure seminoma. False-positive elevations may be associated with hepatic impairment, hepatitis virus infection, or cirrhosis^26^. hCG is a glycoprotein hormone secreted by trophoblastic cells, with small amounts potentially secreted by the pituitary gland. Choriocarcinoma retains the ability to secrete hCG during reproduction and differentiation, leading to markedly elevated levels. Embryonal carcinoma, possessing multidirectional differentiation potential, can see 40%−60% of cases exhibit elevated hCG due to partial differentiation towards trophoblastic cells. Furthermore, 10–20% of pure seminomas may show elevated hCG due to the presence of syncytiotrophoblastic cells^27^. However, hCG elevation is not exclusive to malignancy and can be influenced by various factors, including benign conditions like hypogonadism – which causes compensatory increases in pituitary FSH and LH, both potentially cross-reacting in immunoassays and causing falsely elevated hCG^28^. Cannabis use or heterophilic antibodies in the blood^29^ can also lead to elevated hCG^30,]^ alongside laboratory factors such as cross-contamination or differing assay methodologies.In addition to traditional biomarkers, an increasing number of novel markers^31^ have emerged and are expected to be applied in predictive model studies in the future.

This study has a retrospective design and only enrolled patients who underwent surgery, including radical orchiectomy and testis-sparing surgery.In addition, we acknowledge that inter-observer variability of ultrasound features was not analyzed in this study. There may be some variability among different observers, which leads to a certain degree of subjectivity in ultrasound results. In future prospective studies, we will fully take these important points into account to further improve the quality and reliability of our research.

## Conclusion

In summary, our study analyzed 12 parameters encompassing clinical data, imaging, and laboratory findings from testicular tumor patients. Statistical analysis identified five independent discriminative factors: tumor size, boundary, AFP, hCG, and CDFI. Based on these, both static and dynamic visual nomogram prediction models were developed. Evaluation and validation using ROC curves, calibration curves, and DCA demonstrated favorable predictive performance. The overall model AUC showed substantial improvement over any single indicator, enhancing the ability to identify more patients suitable for Testis-Sparing Surgery (TSS) or active surveillance. Compared to previous studies, our model demonstrates advantages including a larger sample size, higher accuracy, incorporation of widely accessible parameters, and user-friendly visualization.Future studies should include more research centers and incorporate additional variables to update and optimize the existing model.

## Data Availability

The datasets used and/or analysed during the current study available from thecorresponding author on reasonable request.
